# 

**Published:** 1846-01

**Authors:** 


### CONTENTS OF N° XLI

OF THE

### British and Foreign Medical Review.

JANUARY, 1846.

### PART FIRST.--Analytical and Critical Reviews.

PAGE

ART. I.—1. The Sixth Annual Report of the Registrar-General of Births, Deaths, and Marriages, in England, 1845 . . . . . 1

2. Contributions to Vital Statistics: being a Development of the Rate of Mortality and the Laws of Sickness; from original and extensive data, procured from Friendly Societies, showing the Instability of Friendly Societies, "Odd Fellows," "Rechabites," &c., with an Inquiry into the Influence of Locality on Health. By F. G. P. NEISON, F.L.S., &c. . . ib.

3. Facts connected with the Social and Sanitary Condition of the Working Classes in the City of Dublin, with Tables of Sickness, Medical attendance, Deaths, Expectation of Life, &c. &c.; together with some Gleanings from the Census Returns of 1841. By THOMAS WILLIS, F.S.S. . . ib.

4. On the Duration of Life among the Families of the Peerage and Baronetage of the United Kingdom. By WILLIAM A. GUY, M.B. Cantab., &c. &c. ib.

ART. II.—1. Dr. J. L. Schönlein's, Professor in Zürich, Allgemeine und Specielle Pathologie und Therapie, &c. &c. In Vier Theilen . . 20

The General and Special Pathology and Therapeutics of Dr. J. L. Schönlein, Professor in Zürich. Edited by several of his Class, from Notes taken at his Lectures.

2. Schönlein's Klinische Vorträge in dem Charité Krankenhause zu Berlin. Redigirt und herausgegeben von Dr. L. GÜTERBOCK . . . ib.

Dr. Schönlein's Clinical Discourses in the Charité Hospital at Berlin. Taken and edited by Dr. L. GÜTERBOCK.

3. Dr. Schönlein als Artz und klinischer Lehrer aus der Schilderung der Dr. Güterbock; einer unabweisbaren Kritik unterworfen von Dr. LEHRS und Dr. SCHARLAU . . . . . . ib.

An unimpeachable Critique on Dr. Schönlein, as Physician and Clinical Lecturer, &c. &c. By Dr. LEHRS and Dr. SCHARLAU.

ART. III.—On Diseases of the Liver. By GEORGE BUDD M.D. F.R.S. . 39

4298

PAGE

ART. IV.—1. Odontography; or a Treatise on the Comparative Anatomy of the Teeth; their Physiological Relations, &c. By RICHARD OWEN, F.R.S. &c. 62

2. Report on the Microscopic Structure of Shells. Part I. By WILLIAM B. CARPENTER, M.D. F.R.S. . . . . . ib.

3. Observations on the Structure of the Shells of Molluscous and Conchiferous Animals. By JAMES SCOTT BOWERBANK, F.R.S. &c. . . ib.

ART. V.—A Treatise on the Diseases and Special Hygiene of Females. By COLOMBAT DE L'ISERE. Translated from the French, with Additions, by CHARLES D. MEIGS, M.D. &c. . . . . 83

ART. VI.—Medical and Physiological Problems, being chiefly Researches for Correct Principles of Treatment in Disputed Points of Medical Practice. By WILLIAM GRIFFIN, M.D., Member of the Royal College of Surgeons in Edinburgh, one of the Physicians to the County of Limerick Infirmary, &c.; and by DANIEL GRIFFIN, M.D. . . . . 92

ART. VII.—Chemistry, Meteorology, and the Function of Digestion, considered with Reference to Natural Theology. By WILLIAM PROUT, M.D. F.R.S. . 109

ART. VIII.—1. Traité des Maladies des Articulations; accompagné d'un Atlas de 16 Planches. Par A. BONNET . . . . . 124

A Treatise on Diseases of the Joints; with an Atlas of 16 Plates. By A. BONNET.

2. Researches pour servir à l'Histoire des Tumeurs Blanches. Par M. RICHET ib.

Researches intended to elucidate the History of White Swellings. By M. RICHET.

3. De la Résection de la Tête du Fémur. Par M. BONINO . . ib.

On the Resection of the Head of the Femur. By M. BONINO.

4. De la Coxalgie. Par M. le Docteur J. G. MAISONNEUVE . . ib.

On Coxalgia. By J. G. MAISONNEUVE, M.D.

5. Recherches sur le Mal Vertébral de Pott. Mémoire couronné par le Conseil der Hôpitaux. Par le Docteur TAVIGNOT . . . ib.

Researches on Pott's Disease of the Spine. By Dr. TAVIGNOT; being a Memoir to which a Prize was awarded by the Council of Hospitals of Paris.

6. Maladie des Articulations Costo-chondrales et Costo-vertébrales, avec ou sans Ramollissement Tuberculeux et Nécrose des Os du Rachis. Par A. TOULMOUCHE, D.M. . . . . . . . ib.

On Disease of the Articulations of the Ribs, with or without Tubercular Softening and Necrosis of the Vertebræ. By Dr. A. TOULMOUCHE.

ART. IX.—Untersuchungen über Entstehung des Krankheitsgenius, &c. &c. Von Dr. MARTIN GEIGEL, &c. . . . . . 145

Researches into the Origin of the Morbid State, &c. By Dr. M. GEIGEL.

ART. X.—Fruits and Farinacea the proper Food of Man; being an attempt to prove from History, Anatomy, Physiology, and Chemistry, that the original, natural, and best Diet of Man is derived from the Vegetable Kingdom. By JOHN SMITH . . . . . . 148

ART. XI.—Traité philosophique et clinique d'Ophthalmologie, basé sur les principes de la Thérapeutique Dynamique. Par M. F. ROGNETTA . . 158

A Philosophical and Clinical Treatise on Ophthalmology, based on the principles of the Dynamic Therapeutics. By F. ROGNETTA, M.D.

ART. XII.—A View of the Formation, Discipline, and Economy of Armies. By the late ROBERT JACKSON, M.D. . . . 166

PAGE

Art. XIII.—A practical Treatise on Inflammation, Ulceration, and Induration of the Neck of the Uterus: with Remarks on the value of Leucorrhœa and Prolapsus Uteri as Symptoms of Uterine Disease. By J. H. Bennet, M.D. . 174

Art. XIV.—De Tenotomia Talipedibus applicata. Commentatio quam scripsit. Chr. Weis . . . . . . . 182

A Treatise on Tenotomy as applied to Club-foot. By C. Weis.

Art. XV.—Beiträge zur Medizin, Chirurgie und Ophthalmologie Von Chr. Conr. Wuth . . . . . . . 186

Contributions to Medicine, Surgery, and Ophthalmology. By C. C. Wuth, M.D.

Art. XVI.—'The Lost Senses'—Deafness. By John Kitto, D.D. . . 188

Art. XVII.—A practical Treatise on Healthy Skin: with Rules for the Medical and Domestic Treatment of Cutaneous Diseases. By Erasmus Wilson, F.R.S. 198

Art. XVIII.—1. Saggio Illustrativo le Tavole della Statistica Medica delle Maremme Toscane. Compilata per ordine di S.A.R. il Gran-Duca di Toscana. Da Antonio Salvagnoli-Marchetti . . . . . 205

Essay illustrating the Tables of the Medical Statistics of the Tuscan Maremma. Compiled by order of H.R.H. the Grand Duke of Tuscany. By Antonio Salvagnoli-Marchetti.

2. Versuch einer Medicinischen Topographie und Statistik von Berlin. Von Dr. H. Wollheim, praktischem Arzte, Wundarzte und Geburtshelfer. Mit einem Vorworte von Dr. J. L. Casper . . . . ib.

Medical Topography and Statistics of Berlin. By Dr. H. Wollheim, with a Preface by Dr. J. L. Casper.

Art. XIX.—The Nature and Treatment of Gout. By W. H. Robertson, M.D. 215

Art. XX.—The Nature and Treatment of Cancer. By W. H. Walshe, M.D. 218

Art. XXI.—1. Organon der Heilkunst. Von Samuel Hahnemann . . 225

2. Fragmenta de Viribus Medicamentorum positivis, sive in sano corpore humano observatis. A Samuele Hahnemann, M.D. . . . ib.

3. Samuelis Hahnemanni Materia Medica Pura, sive Doctrina de medicamentorum viribus in corpore humano sano observatis, e Germanico sermone in Latinum conversa. Conjunctis studiis ediderunt Dr. Stapf, Dr. G. Gross et E. G. à Brunnow . . . . . . ib.

4. Die Chronischen Krankheiten, ihre eigenthümliche Natur und homöopathische Heilung. Von Dr. Samuel Hahnemann . . ib.

5. Pharmacopœia Homœopathica. Edidit T. F. Quin . . ib.

6. Homœopathy Unmasked; being an Exposure of its principal Absurdities and Contradictions: with an Estimate of its recorded Cures. By Alexander Wood, M.D. . . . . . . . ib.

7. An Introduction to the Study of Homœopathy. Edited by J. J. Drysdale, M.D. and J. R. Russel, M.D. . . . . . . ib.

8. An Inquiry into the Homœopathic Practice of Medicine. By W. Henderson, M.D. . . . . . . . . ib.

9. Homœopathic Domestic Medicine. By J. Laurie, M.D. . . ib.

Art. XXII.—An experimental Inquiry into the Pathology and Treatment of Asphyxia. By John E. Erichsen . . . . . 265

Art. XXIII.—Lectures on the Theory and Practice of Surgery. By the late Abraham Colles, M.D., &c. Edited by Simon M'Coy, Esq. F.R.C.S.I. . 271

### PART SECOND.—Bibliographical Notices.

PAGE

ART. I.—Observations on the Nature and Treatment of the more important Diseases of the Nervous System. By a Physician . . . 273

ART. II.—Rapport Annuel sur les Travaux de la Société Médicale du Canton de Genève. Pour l'année 1843. Par le Docteur MARC D'ESPINE . 274

Annual Report of the Medical Society of the Canton of Geneva. By Dr. MARC D'ESPINE.

ART. III.—Das Kindbettfieber in nosologischer und therapeutischer Beziehung. Von Dr. C. T. LITZMANN . . . . . 275

The Nature, History, and Treatment of Childbed Fever. By Dr. LITZMANN.

ART. IV.—An Act for the Regulation of the Care and Treatment of Lunatics; with explanatory Notes and Comments. Edited by FORBES WINSLOW, M.D. . 276

ART. V.—Illustrations of Modern Memerism from Personal Investigation. By JOHN FORBES, M.D. F.R.S. . . . . . 277

ART. VI.—Notice sur les Variations du Poids des Prisonniers soumis au Régime Pénitentiare. Par le Docteur MARC D'ESPINE . . . 278

On the Variations in the Weight of Prisoners submitted to the Penitentiary System. By Dr. MARC D'ESPINE, &c.

ART. VII.—Sopra Gentile da Fuligno, Medico Illustre del Secola XIV. Discorso Storico-critico del Dottor GIUSEPPE GIROLAMI . . 279

A critical and historical Discourse regarding Gentili da Fuligno, a celebrated Physician of the 14th century. By Dr. G. GIROLAMI.

ART. VIII.—Beiträge zur Erkenntniss und Heilung der Spinal-Neurosen. Von Dr. GEORG. HIRSCH . . . . . . . 280

Contributions to the Diagnosis and Treatment of Spinal Neuroses. By Dr. G. HIRSCH, &c.

ART. IX.—Ueber eine bis jetzt noch nicht näher beschriebene Drüse im innern der Zungenspitze. Von A. NÜHN . . . . 281

On a Gland, hitherto not accurately described, in the interior of the tip of the Tongue. By A. NÜHN, M.D.

ART. X.—A Clinical Introduction to the Practice of Auscultation, and other Modes of Physical Diagnosis; intended to simplify the Study of the Diseases of the Lungs and Heart. By H. M. HUGHES, M.D. . . . ib.

ART. XI.—The History of the Middlesex Hospital during the first century of its existence. By ERASMUS WILSON, F.R.S. . . . 282

ART. XII.—Elementa Physiologiæ specialis Corporis Humani. Auctore AUGUSTO ARNOLDO SEBASTIAN . . . . . ib.

ART. XIII.—An Inaugural Address, delivered at the opening of the Norfolk and Norwich Hospital Museum, Sept. 1845. By J. G. CROSSE, M.D. F.R.S. . 283

Books received for Review . . . . . . 284

